# Enhancing the Stability of 4.6 V LiCoO_2_ Cathode Material via Gradient Doping

**DOI:** 10.3390/nano14020147

**Published:** 2024-01-09

**Authors:** Errui Wang, Xiangju Ye, Bentian Zhang, Bo Qu, Jiahao Guo, Shengbiao Zheng

**Affiliations:** 1 College of Chemistry and Material Engineering, Anhui Science and Technology University, Bengbu 233030, Chinazhangbt@ahstu.edu.cn (B.Z.);; 2 Anhui Province Quartz Sand Purification and Photovoltaic Glass Engineering Research Center, Anhui Science and Technology University, Bengbu 233030, China

**Keywords:** Li-ion batteries, LiCoO_2_, gradient doping, electrochemical

## Abstract

LiCoO_2_ (LCO) can deliver ultrahigh discharge capacities as a cathode material for Li-ion batteries when the charging voltage reaches 4.6 V. However, establishing a stable LCO cathode at a high cut-off voltage is a challenge in terms of bulk and surface structural transformation. O_2_ release, irreversible structural transformation, and interfacial side reactions cause LCO to experience severe capacity degradation and safety problems. To solve these issues, a strategy of gradient Ta doping is proposed to stabilize LCO against structural degradation. Additionally, Ta_1_-LCO that was tuned with 1.0 mol% Ta doping demonstrated outstanding cycling stability and rate performance. This effect was explained by the strong Ta-O bonds maintaining the lattice oxygen and the increased interlayer spacing enhancing Li^+^ conductivity. This work offers a practical method for high-energy Li-ion battery cathode material stabilization through the gradient doping of high-valence elements.

## 1. Introduction

Lithium-ion batteries (LIBs) are the most prevalent energy storage devices and are preferred due to their elevated energy density (>150 Wh/kg) [1,2,3,4]. Among the mainstream cathode materials, LiCoO_2_ (LCO) is one of the most widely used for lithium-ion batteries (LIBs) and is preferred in consumer electronics due to its high tap density [5,6]. It has an ultrahigh discharge capacity greater than 220 mAh/g and a gravimetric energy density beyond 850 Wh/kg at a working cut-off voltage of 4.6 V [7,8]. However, because of interfacial side reactions, irreversible structural transformation, and O_2_ release, LCO experiences substantial capacity loss and presents safety concerns at such high voltages [9,10,11,12]. When the voltage is higher than 4.5 V, accompanied by an adverse phase transformation from O3 to H1-3 via Li^+^ rearrangement and lattice shrinkage along the c-axis direction, it results in pernicious structural evolution and particle fragmentation [13,14,15]. Meanwhile, because of the overlap in energy levels between the O 2p and Co 3d orbitals, oxygen atoms within the lattice, particularly those near the surface, tend to be released as O_2_, which triggers the surface structural degradation of LCO and promotes electrolyte decomposition, forming a thick cathode electrolyte interphase (CEI) layer [16,17,18]. Due to these reasons, its electrochemical performance deteriorates at a high voltage, and this significantly restricts its practical application.

Numerous modification techniques have been suggested in order to address these problems and enhance the cycle stability of LiCoO_2_ beyond 4.5 V. The most promising and successful conventional techniques for improving both the structural robustness and the electrochemical efficacy of LiCoO_2_ under elevated voltages (>4.5 V) is element doping [8,19,20,21,22]. Hetero-elements can regulate the atomic-level lattice and fundamental physicochemical characteristics of materials [23,24]. Huang et al. reported that LiCoO_2_ with Mg doped into the Li layer and Mg-pillared LiCoO_2_ exhibit outstanding capacity retention, reaching 84% over 100 cycles at a rate of 1 C within the voltage range of 3.0–4.6 V [8]. Additionally, Mg-doped LiCoO_2_ can effectively alleviate the lattice strain [16]. Myung et al. demonstrated that Al-doped LiCoO_2_ not only inhibits phase transition but also inhibits transition metal dissolution [25]. In addition, in multiple-element co-doping, the applied elements were found to synergistically improve the electrochemical characteristics of LiCoO_2_, including Mg-Cu [26], Al-La [27], Ca-P [17], Ti-Mg-Sb [7], and Al-Mg-Ti [13,28]. Liu et al. demonstrated that the double-doping of LiCoO_2_ with Ca-P mitigated LiCoO_2_ irreversible structural transformation, largely enhancing both cycling stability and rate performance [17]. Zhang et al. proposed a synergistic approach to enhance 4.6 V LiCoO_2_ cycle performance using tri-element co-doping with Ti, Mg, and Al [13]. Mg and Al doping inhibited the irreversible structural transition at 4.6 V. Furthermore, the Ti element was highly concentrated both on the surface and at grain boundaries, which could prevent interface side reactions, as well as surface oxygen release at high voltages. Consequently, after 100 cycles, the doped LiCoO_2_ showed an 86% capacity retention at 4.6 V. Contrasting the significant structural deterioration of LiCoO_2_ and the lattice oxygen release from it at high voltage, multiple-element doping, through a synergistic effect, can effectively stabilize LiCoO_2_ bulk and surface structure, which is beneficial in improving LiCoO_2_ electrochemical performance. Uncertainty exists regarding the multi-element co-doping synergistic process. Simultaneously, the process of alteration is intricate, making it challenging to attain structural consistency and electrochemical performance repeatability. It was established that gradient element doping is a successful strategy for improving the cycle stability of cathode materials [29]. In gradient doping, the dopant concentrations differ from the surface to the bulk, which is advantageous as it can improve the structural stabilities of both [18]. In addition, high-valence elements (M) are favorable candidates for gradient doping, as the lattice oxygen of a layered oxide cathode material can be stabilized by the high bond energy of M-O [30,31,32]. For example, Sun et al. explained the mechanism of Ta doping to improve the stability of high-nickel cathode materials and found that the doping of high-valence elements changed the growth of primary particles, which acted as an atomic support for secondary particles and prevented the collapse of the material structures during charging and discharging [31]. Huang et al. designed an experiment to test the surface enrichment with Ta. Ta doping changed the atomic structure and local charge distribution on the surface, and the Ta-enriched layer on the surface also inhibited side reactions, thus improving the cycling performance [32]. However, few studies have been conducted to explore the inherent impact of gradient Ta doping on LiCoO_2_.

In order to enhance the structural robustness and electrochemical capabilities of LiCoO_2_, gradient Ta doping was carried out in this study. Subsequently, several approaches were used to determine the mechanism underlying the resulting improvement. Structure evolution and the anion redox reaction were investigated using in situ XRD and XPS. Thus, at high voltages, the strong Ta-O bond after gradient Ta doping could efficiently suppress interfacial side reactions and surface oxygen release in the lattice, stabilizing the structure of LiCoO_2_ during extended cycling. The optimized cathode material with 1.0 mol% Ta doping exhibited outstanding stability. It maintained a capacity of 88% after 150 cycles at a rate of 0.5 C. Additionally, it demonstrated superior rate performance, attaining a capacity of 165.1 mAh/g at a rate of 5 C within the voltage range of 3.0–4.6 V.

## 2. Materials and Methods

### 2.1. Material Synthesis

The preparation of cathode materials involved the synthesis of unmodified LiCoO_2_ (LCO) and LiCoO_2_ with gradient Ta doping, accomplished through a solid-state reaction method, as follows. A stoichiometric ratio of Li_2_CO_3_ (99.5%, Shanghai Maclin Biochemical Technology Co., Ltd., Shanghai, China) and Co_3_O_4_ (99%, Shanghai Maclin Biochemical Technology Co., Ltd., Shanghai, China) was uniformly ground for 40 min within a mortar made of agate and subsequently calcined for 15 h at 950 °C. Once subjected to a heating rate of 5 °C in ambient air, the material was cooled to room temperature to yield the final product. Gradient-Ta-doped LCO samples were created using the same synthesis technique with varying doping contents of 0.5, 1, and 2 mol% Ta by adjusting the ratios of Li_2_CO_3_, Co_3_O_4_, and Ta_2_O_5_ (99%, Shanghai Maclin Biochemical Technology Co., Ltd., Shanghai, China) and were named Ta_0.5_-LCO, Ta_1_-LCO, and Ta_2_-LCO, respectively.

### 2.2. Structure Characterization

XRD analysis was employed to examine the crystal structure of both bare and doped samples (Bruker D8 Advance, Karlsruhe, Germany). XPS was employed to examine the elemental oxidation states at the surface (PHI Quanteral II, Chigasaki, Japan). SEM (SU80020) and element diffraction spectroscopy (EDS) were carried out to determine each material’s morphology and elemental distribution. For the surface lattice oxygen activity test, coin cells were charged to 4.6 V and then removed from the Ar glove box (H_2_O and O_2_ content below 0.1 ppm). The cathode electrode was washed with dimethyl carbonate (DMC) to remove the remaining electrolyte, and, subsequently, the cathode was transferred to a sealed bag and then analyzed by XPS. The samples’ microstructure was analyzed using TEM (JEM-2100F, Tokyo, Japan). During transfer to the XPS and XRD equipment, the samples were briefly exposed to air, which had a negligible effect on the test results. Similarly, the preparation of the TEM test samples was also carried out in the Ar glove box (H_2_O and O_2_ contents below 0.1 ppm). The assembly of CR2032 coin-type cells followed the methodology outlined in a prior study [33].

### 2.3. Electrochemical Measurements

For the cathodes, the active material consisted of LiCoO_2_ (80 wt%), along with carbon black (10 wt%) and pre-dissolved PVDF (10 wt%) in *N*-methyl pyrrolidone. The components were blended in an 8:1:1 ratio to create a slurry that was uniformly applied to Al current collectors (20 μm). Subsequently, the aluminum current collectors underwent a vacuum drying process lasting 12 h at 120 °C within a vacuum oven, and then the cathodes were rolled into thin films with an active material mass loading of approximately 3 mg/cm^2^. The cathode cut piece was transformed into a circular piece with a diameter of 11 mm. Lithium plates (thickness of 2 mm) were used for the negative electrode and a polypropylene (Celgard 2400, Shenzhen, China) microporous membrane for the diaphragm; the electrolyte was 1.2 M LiPF_6_ in ethylene carbonate and dimethyl carbonate, with a volume ratio of 3:7. The 2023-type coin cells (stainless-steel materials, Shenzhen Kejing Star Technology Co., LTD, Shenzhen, China) were assembled in a glove box filled with argon. The cells underwent testing within a voltage range from 3.0 to 4.6 V at different rates. The electrochemical cycling performances of LCO and Ta_1_-LCO were evaluated at 0.5 C (1C = 274 mAh/g) and 25 °C. The relevant electrochemical performance tests were conducted by utilizing a battery test technology from NEWARE. For in situ XRD, a specially designed in situ cell with an X-ray transparent beryllium window was used, and each pattern (from 15° to 50°) was collected every 13 min while the cell was operated at a current of 0.1 C. GITT curves of LCO and Ta_1_-LCO in the first charges were obtained with a 0.1 C charge/discharge current density and a time interval of 2 h.

## 3. Results and Discussion

### 3.1. Structural Analysis

**Figure 1:**
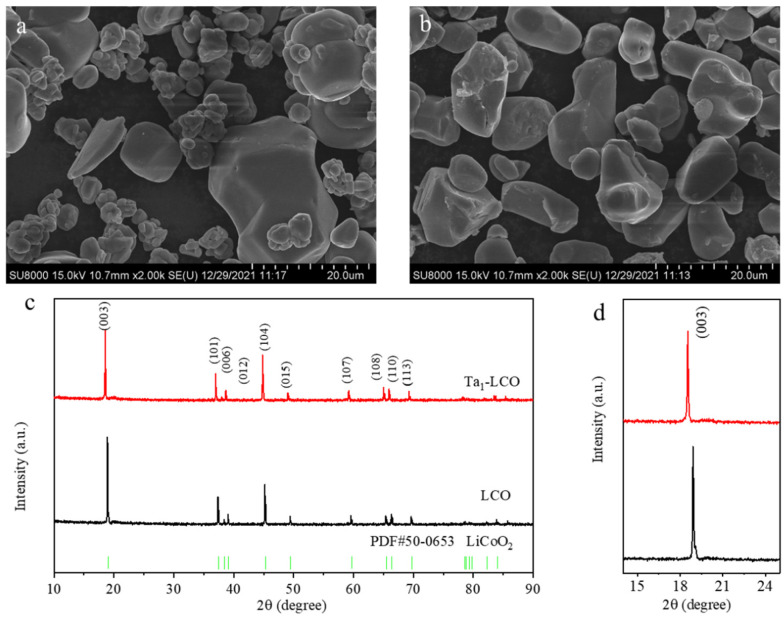
(**a**,**b**) SEM images of LCO and Ta_1_-LCO particles. (**c**) XRD patterns of LCO and Ta_1_-LCO particles, corresponding to the standard card of LCO (PDF#50-0653). (**d**) The enlarged view of the (003) peaks in the 2θ range of 14–25°.

The morphology and particle distribution of LCO and Ta_1_-LCO (as an example of gradient-Ta-doped LCO) are crucial to attaining the desired electrochemical performance of Li-ion batteries. Overall, the partial particle size was small, as demonstrated for LCO in Figure 1a,b, and certain small particles had the potential to aggregate into larger particles. However, Ta_1_-LCO showed a homogeneous particle size and a smooth surface, which indicated that doping with a small amount of Ta might contribute to crystal growth and uniform particle size distribution. LCO and Ta_1_-LCO were analyzed using XRD to examine the impact of gradient Ta doping on the crystal structure of LCO cathode materials. The XRD patterns of the LCO and Ta_1_-LCO particles are shown in Figure 1c. The results showed that all the diffraction spectra of the LCO and Ta_1_-LCO particles could be indexed within the α-NaFeO_2_ structure (space group R$\bar{3}$m), with no other impurity peaks being observed, indicating that pure-phase cathode materials had been obtained [15,34,35,36]. The LCO particles still consisted of just one phase after gradient Ta doping. Highly crystalline layered structures were all well developed, as demonstrated by the clear split of the (006)/(012) and (018)/(110) peaks of the LCO and Ta_1_-LCO particles [37,38,39]. However, the (003) diffraction peak of Ta_1_-LCO exhibited a downward shift in 2θ, primarily attributed to the expansion of the (003) plane spacing, as illustrated in Figure 1d. The XRD results imply that Ta^5+^ entered the lattice of LCO successfully, and its amount increased in the interlayer distance, which was beneficial in improving Li^+^ transport in Ta_1_-LCO.

**Figure 2:**
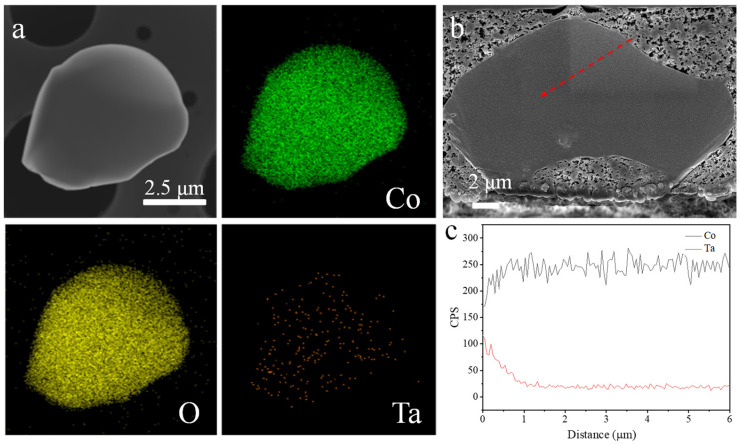
(**a**) SEM and EDS mapping of the elements Ta, Co, and O on the surface of Ta_1_-LCO. (**b**) Cross-sectional SEM image of Ta_1_-LCO. (**c**) EDS line scanning of Ta and Co along the red arrow in (**b**).

The distribution of Ta in Ta_1_-LCO was further examined by employing energy-dispersive X-ray energy spectroscopy (EDS) mapping. Ta was successfully doped onto the surface of Ta_1_-LCO, as demonstrated in Figure 2a, and its uniform distribution suggested that Ta did not affect the Co and O element distribution on the LCO surface. To confirm the doping element Ta dispersion in the bulk regions of the Ta_1_-LCO particles, an EDS line scanning analysis was utilized to explore the elemental concentrations from the surface to the central regions of particle cross sections (Figure 2b). Figure 2c presents the EDS spectra collected from the surface to the central regions of a Ta_1_-LCO particle cross section. The concentration of Ta gradually increased from the interior to the exterior of Ta_1_-LCO, which indicated that the bulk content of Ta was relatively low, and a high content of Ta was present on the surface of Ta1-LCO. Meanwhile, in comparison to the bulk region, the surface region had a lower concentration of Co. Thus, the gradient doping of Ta in Ta_1_-LCO was confirmed. Consequently, the surface regions in direct contact with the electrolyte would limit the occurrence of side reactions at the interface between the electrode and the electrolyte.

### 3.2. Electrochemical Performance

**Figure 3:**
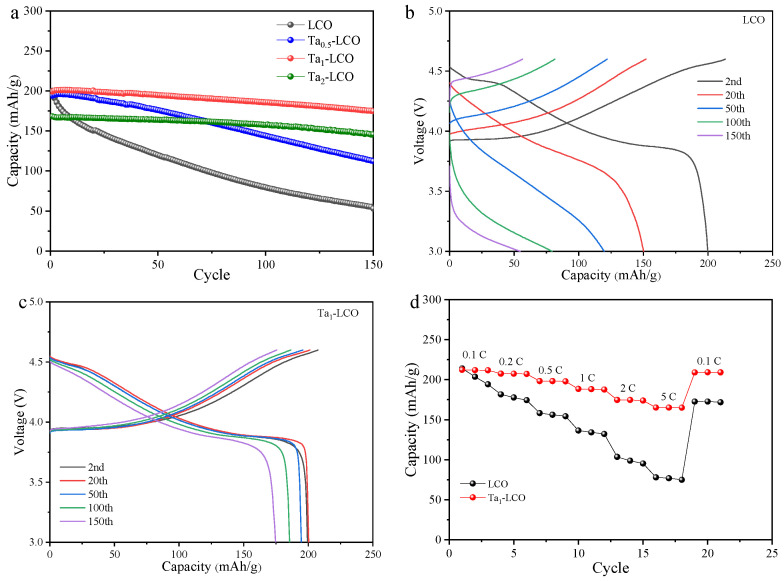
(**a**) Cycling performance of all batteries at 0.5 C. Charge/discharge curves of (**b**) LCO and (**c**) Ta_1_-LCO at different cycles. (**d**) Rate performance of LCO and Ta_1_-LCO.

The electrochemical performances of all the batteries were assessed, using 2023-type coin cells in the 3.0–4.6 V voltage range with lithium foil anodes, to determine the ideal Ta doping level. An active substance was used to obtain either LCO or Ta_n_-LCO working electrodes with varying indices (n = 0.5, 1, and 2). This active material was combined with carbon black and a PVDF binder. The mass ratio of these components was 8:1:1. The active material ultimate electrode loading was approximately 3 mg/cm^2^. The electrolyte was l.2 M LiPF_6_ in ethylene carbonate and dimethyl carbonate, with a volume ratio of 3:7. The electrochemical cycling performances of LCO and Ta_1_-LCO were evaluated at 0.5 C and 25 °C. The obtained results indicated superior electrochemical performance for Ta_1_-LCO, as illustrated in Figure 3a. After 150 cycles, Ta_1_-LCO showed an 88% capacity retention and a 174.8 mAh/g capability for release, whereas LCO and Ta_o.5_-LCO exhibited low capacity retentions of only ~26% and 57.8%, respectively. Moreover, Ta_2_-LCO had an 87% capacity retention as well as a reduced initial capacity of discharge (168.1 mAh/g). These findings showed that although insufficient Ta doping can only marginally increase cycle stability, excessive Ta doping can decrease the reversible capacity. Additionally, the initial Coulombic efficiency of the electrode material could be significantly improved after gradient Ta doping. Figure 3b,c shows the LCO and Ta_1_-LCO charge/discharge curves for various cycles. Ta_1_-LCO showed first a Coulomb efficiency of 93.6%, much higher than that of LCO. Ta_1_-LCO showed a greater initial Coulombic efficiency primarily because of its stable crystal structure and mild side interactions on the surface. In addition, while Ta_1_-LCO demonstrated a specific capacity of 218.3 mAh/g, LCO exhibited a capacity of 213.1 mAh/g at 0.1 C during the initial cycle. This suggested that gradient Ta doping did not exert a noticeable influence on the reversible capacity in the first cycle.

The voltage decay that resulted in additional energy density fading might have been caused by structural transformation. Upon cycling, the voltage platform and capacity of LCO sharply declined, indicating that LCO suffered a severe structural degradation. However, Ta_1_-LCO exhibited a low capacity fading and no obvious electrochemical polarization. The rate performance of Ta_1_-LCO and LCO was further tested using current densities of 0.1 C, 0.2 C, 0.5 C, 1 C, 2 C, and 5 C within a 3.0 V–4.6 V cut-off voltage range to assess the practical applicability of LCO in consumer electronic goods. Benefitting from the expansion of interlayer distance, a superior rate performance of Ta_1_-LCO was obtained, according to Figure 3d. With just a 36.4% capacity retention rate, the discharge capacity of LCO dropped from 213.1 mAh/g at 0.1 C to 78 mAh/g at 5 C. For Ta_1_-LCO, the discharge capacity remained at 165.1 mAh/g at 5 C, and capacity retention could be as high as 77.8%. To some extent, the outstanding rate performance of Ta_1_-LCO suggested that gradient Ta doping improved the diffusion rate of Li^+^, which could lessen surface impedance and polarization. The excellent electrochemical performance of Ta_1_-LCO suggested that gradient Ta doping could improve the Li^+^ diffusion kinetics in the LCO electrode and its structural stability.

Reaction kinetics are a key factor affecting an electrode electrochemical performance. The galvanostatic intermittent titration technique (GITT) was utilized to investigate the impact of Ta doping on the Li^+^ diffusion coefficient (D_Li+_, determined by the systematic error). The Li^+^ diffusion coefficient (D_Li+_) in both LCO and Ta_1_-LCO were determined through the application of the following Equation (1) based on the GITT results [40,41]:

(1) $$D_{{Li}^{+}}=\frac{4}{\pi \tau }{\left(\frac{m_{B}V_{M}}{M_{B}S}\right)}^{2}{\left(\frac{∆E_{S}}{{∆E}_{\tau }}\right)}^{2}$$

**Figure 4:**
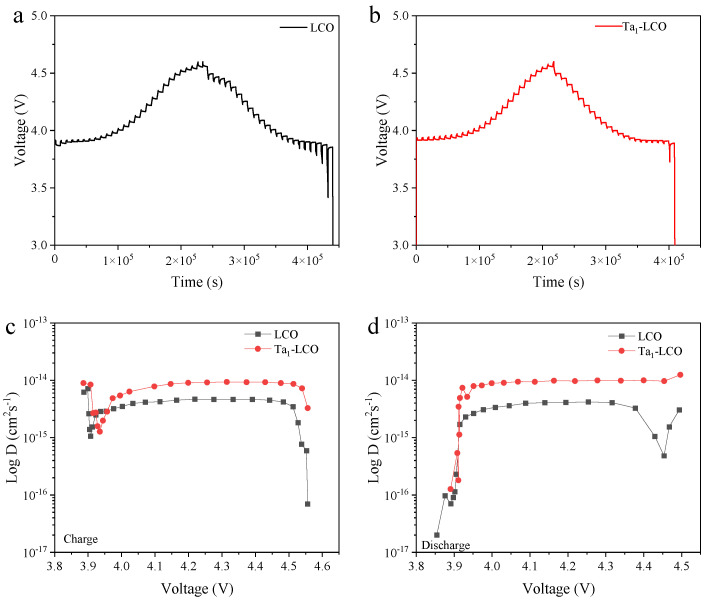
Charge/discharge curves of GITT for (**a**) LCO and (**b**) Ta_1_-LCO during the first cycle. The calculated D_Li+_ of (**c**) LCO and (**d**) Ta_1_-LCO during the first cycle.

In this formula, the molecular weight *(m_B_*) and mass (*M_B_*) of the active ingredient are shown. The molar volumes of LCO and Ta_1_-LCO are denoted by *V_M_*. *S* represents the active surface area of LCO and Ta_1_-LCO, which was 0.85 m^2^/g and 0.14 m^2^/g for LCO and Ta_1_-LCO, respectively, as obtained from the BET testing. As a result, the Ta_1_-LCO electrode exhibited a lower electrochemical polarization than the LCO electrode (Figure 4a,b), which might be due to the reduced interfacial side reactions and restrained structural transformation, as well as to improved interfacial charge diffusion. Moreover, as shown in Figure 4c,d, Ta_1_-LCO exhibited a higher D_Li+_ value than LCO throughout the initial charge/discharge procedure. Thus, it was confirmed that gradient Ta doping could significantly improve the D_Li+_ in Ta_1_-LCO, which corresponded to the rate performance outcome.

### 3.3. Structural Evolution

**Figure 5:**
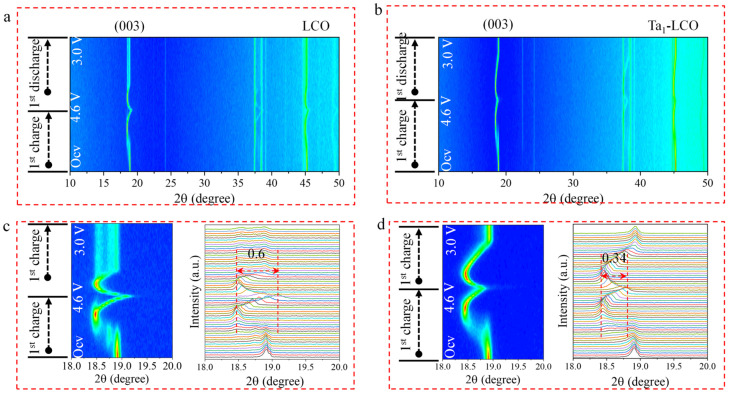
In situ XRD analysis of the first cycles of (**a**) LCO and (**b**) Ta_1_-LCO; evolution of the (003) peak and the corresponding contour plot of (**c**) LCO and (**d**) Ta_1_-LCO.

A deeper look into the enhanced electrochemical performance following gradient Ta doping, the structural stability of the LCO and Ta_1_-LCO cathode materials, and in situ XRD measurements of the structural transition of the LCO and Ta_1_-LCO cathode materials are shown Figure 5. This further illustrates the in situ XRD patterns of the Ta_1_-LCO and LCO cathode materials during the initial charge/discharge operation, ranging from 3.0 to 4.6 V at 0.2 C in the first cycle. The corresponding 2D contour plots are presented in Figure 5a,d. With the exception of a few peaks caused by the test mold (Be) and current collector (Al), all peaks were associated with the LCO and Ta_1_-LCO cathode materials [42]. The accompanying enlarged 2D contour plots of the (003) peaks of the LCO and Ta_1_-LCO cathode materials are shown in Figure 5c,d. The degree of the individual (003) peak shifts indicated how the lattice parameter for the LCO cathode materials changed in an evident manner along the c-axis during the charge/discharge process. The LCO peak (003) shifted gradually towards a reduced diffraction angle, which was integral to the increase in charging voltage during the charge process under 3.9 V; this was attributable to a single-phase solid-solution reaction [18]. However, there is a clear splitting in the LCO (003) peak, which could be attributed to a two-phase reaction. A two-phase reaction often results in greater lattice mismatch, which puts LCO under a great internal strain. The H1-3 phase of the LCO sample was clearly visible when charging over 4.55 V, which caused the transition metal plate to slip irreversibly and shrink abruptly along the c-axis [43]. Although the LCO and Ta_1_-LCO electrodes exhibited a similar lattice evolution in the first charging process, they presented different lattice variation values, as reflected by their respective (003) peak shifts [39]. Ta_1_-LCO exhibited a weak (003) peak shift of 0.34° compared to that of 0.36° for LCO. Hence, compared to Ta_1_-LCO, LCO will accrue higher residual stress during long-term cycling, which will cause great structural degradation and particle breaking. Moreover, the (003) peak of Ta_1_-LCO almost reversibly shifted back to its initial position, and new peaks did not appear during the subsequent procedure of discharge to 3.0 V, which indicated that Ta_1_-LCO showed high structural reversibility. It is evident that gradient Ta doping suppressed the electrode structural deterioration and increased its structural stability by obstructing an unfavorable phase transition at the deeply delithiated state. Therefore, the in situ XRD results indicated that Ta gradient doping can suppress severe structural transitions and improve the cycling stability. In addition, the electrochemical behaviors of LCO and Ta_1_-LCO are consistent with the results of the in situ XRD investigations.

**Figure 6:**
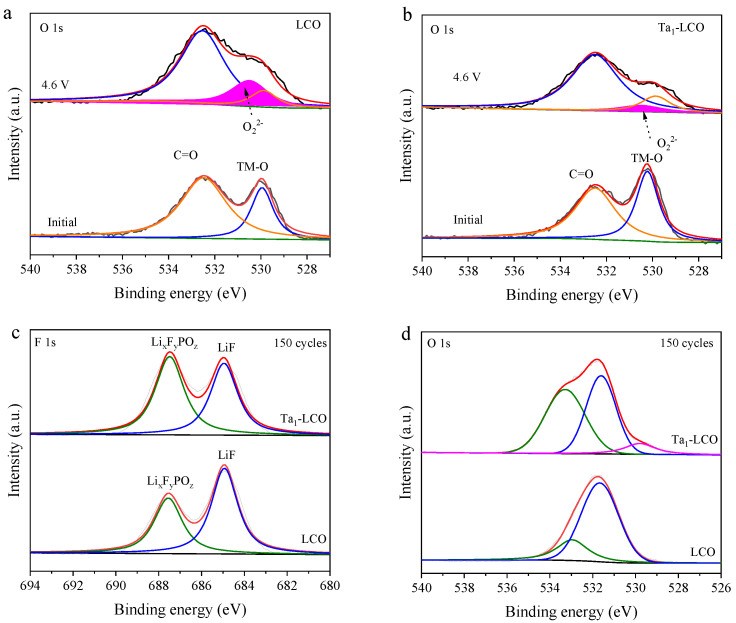
The O 1s XPS spectra of the initial and charged electrodes at 4.6 V: (**a**) LCO and (**b**) Ta_1_-LCO. The XPS spectra of (**c**) F 1s and O 1s (**d**) for LCO and Ta_1_-LCO after 150 cycles.

XPS was utilized to examine the initial and charged electrode at 4.6 V in order to examine the impact of gradient Ta doping on surface lattice oxygen activity in the first charge process. XPS of the initial electrodes provided the O 1s spectrum, which had two distinct peaks in the 528–536 eV region. It may be noted that the relative intensity of the lattice oxygen signal, which peaked near 529.7 eV in the O 1s spectrum (Figure 6a,b), was significantly enhanced after gradient Ta doping, implying that a large number of metal–O (Ta-O) bonds formed on the surface [44]. Examining lattice oxygen development, Figure 6a,b shows the O 1s XPS spectra of Ta_1_-LCO and LCO at 4.6 V. A new characteristic peak (O_2_^2−^) at 530.8 eV gradually formed for both materials, with a 4.6 V cut-off charging voltage, indicating that the anion reaction was activated [45]. However, the area of the O_2_^2−^ characteristic peak was significantly reduced after gradient Ta doping, which indicated that oxygen activity was inhibited following gradient Ta doping. The primary cause of this outcome was the strong Ta-O bond, stabilizing the lattice O. The results indicated that the activity of O at 4.6 V was suppressed by the strong Ta-O bond, which was capable of efficiently maintaining the lattice oxygen.

It is acknowledged that LiF, Li_x_F_y_PO_z_, and Li_2_CO_3_ are the interfacial side reaction products of primary cathode materials in lithium-ion batteries. The continuous formation of CEI layers, resulting from the breakdown of LiPF_6_-based electrolytes under high voltages and prolonged cycling, will result in a fast rise in interface impedance and significant capacity fading [46,47], as these interfacial side reaction products are electrical insulators. The development of CEI after 150 electrochemical cycles of the LCO and Ta_1_-LCO electrodes at 4.6 V was examined via XPS in order to determine the impact of gradient Ta doping on the side reactions taking place on the Ta_1_-LCO electrode’s surface during extended cycling. Overall, a clear difference in the F-containing species was observed in the F 1s spectra. In Figure 6c, the two prominent bands at 686.7 and 685.6 eV were attributed to Li_x_F_y_PO_z_ and LiF, respectively [48]. LiF and Li_x_F_y_PO_z_ were identified as the predominant F-containing constituents of the solid–electrolyte interface (SEI) within LiPF_6_-based electrolytes, primarily resulting from the decomposition of LiPF_6_ [49]. The intensity of the LiF peak observed for the LCO electrode surpassed that for the Ta_1_-LCO electrode surface, indicating more pronounced side reactions at the LCO cathode’s electrode/electrolyte interface. Characteristic bonds of C=O (531.5 eV) and C-O (533.5 eV) are present in the O 1s spectra depicted in Figure 6d [44]. The C=O bond in LCO is more pronounced than that in the reference Ta_1_-LCO, a result associated with the inhibition of EC/DEC decomposition within the electrolyte framework on the electrode surface via gradient Ta doping. In addition, the disappearance of the TM-O peak for the LCO electrode surface indicated that the passivation coating caused by the parasitic reaction was too thick to support the XPS detection of the internal LCO structures. On the other hand, the TM-O peaks of Ta_1_-LCO are visible, indicating that, in contrast to the surface of the cycled LCO, the lattice O was well preserved for the cycled Ta_1_-LCO. These findings showed that gradient Ta doping is efficient and can reduce adverse effects between LCO and electrolytes.

**Figure 7:**
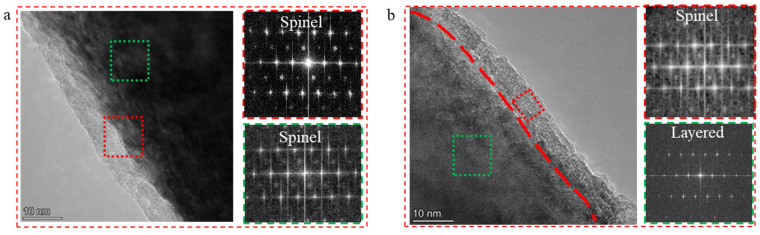
TEM images and the corresponding FFT of (**a**) LCO and (**b**) Ta_1_-LCO after 150 cycles.

To investigate the differences in surface structure evolution between LCO and Ta_1_-LCO during cycling, the cathode materials were examined after 150 cycles using TEM. The TEM images and corresponding FFT images of LCO and Ta_1_-LCO particles are shown in Figure 7. LCO exhibited internal and surface regions that were no longer divided into layers but had a high content of spinel after 150 cycles. In contrast, the Ta_1_-LCO particles inside the zone preserved a well-layered structure, and spinel with only a ~5 nm thickness was present in the surface region. Therefore, it can be further concluded that oxygen release on the surface lattice and structural changes in LCO during battery cycling could be effectively inhibited by Ta doping.

## 4. Conclusions

We reported a unique gradient doping strategy that was developed to improve the electrochemical performance of LiCoO_2_ at 4.6 V. The optimized cathode material (Ta doping = 1.0 mol%) showed a better rate performance of 165.1 mAh/g at 5 C and improved cycle stability, with a capacity retention rate of 88% at 0.5 C after 150 cycles. This was mainly because gradient Ta doping mitigated the irreversible structural transition and stabilized the lattice oxygen in LCO. Overall, this research introduces an elemental gradient doping strategy to aid in the advancement of high-voltage cathode materials, particularly for high-energy batteries.

## Data Availability

Data are contained within the article.
